# *Bacillus subtilis* FZU103 Promotes Growth in *Micropterus salmoides*, Accompanied by Modulation of Gut Microbiota, Enhanced Liver Antioxidants and Digestive Enzyme Activity

**DOI:** 10.3390/microorganisms14010093

**Published:** 2025-12-31

**Authors:** Xu Chen, Hong Zheng, Wenrui Liang, Yinggu Kuang, Xiangzhu Shi, Jinlin Fan, Xucong Lv, Jiacong Deng

**Affiliations:** 1 College of Food and Bioengineering, Fujian Polytechnic Normal University, Fuqing 350300, China; 230827095@fzu.edu.cn (X.C.);; 2 Institute of Food Science and Technology, College of Biological Science and Technology, Fuzhou University, Fuzhou 350108, China; 3 College of Food Science, Fujian Agriculture and Forestry University, Fuzhou 350002, China; 4 Fujian Xinminke Biotech Co., Ltd., Fuzhou 350008, China; 5 Key Laboratory of Eel Aquaculture and Processing of Fujian Province, Fuzhou University, Fuzhou 350108, China

**Keywords:** *Bacillus subtilis* FZU103, *Micropterus salmoides*, growth performance, gut microbiota, digestive enzymes, hepatic function

## Abstract

Probiotics hold great potential in aquaculture, as they can effectively modulate gut microbiota and improve fish health, thereby enhancing farming efficiency. Translating this potential into practical application critically relies on screening high-efficacy probiotic strains. This study evaluated the growth-promoting and health-enhancing effects of probiotic candidates *Lactobacillus rhamnosus* GG (LGG), *Lactobacillus plantarum* FZU310 (LP-FZU310) and *Bacillus subtilis* FZU103 (BS-FZU103) in largemouth bass (*Micropterus salmoides*). After feeding different probiotics for 30 days, the growth, antioxidant, and intestinal enzyme indicators of *M. salmoides* were detected. BS-FZU103 demonstrated superior efficacy among the tested strains, showing significant differences in both specific growth rate (SGR) (*p* < 0.05) and condition factor (CF) (*p* < 0.05). It also markedly enhanced hepatic antioxidant status, elevating superoxide dismutase and glutathione peroxidase activities while reducing malondialdehyde levels by 80%. Improved liver integrity was indicated by significant decreases in serum alanine aminotransferase, aspartate aminotransferase and alkaline phosphatase. Digestively, BS-FZU103 specifically increased intestinal amylase activity by 14.7%, without affecting protease or lipase, suggesting enhanced carbohydrate digestion. 16S rRNA sequencing revealed BS-FZU103 remodeled gut microbiota, increasing *Proteobacteria* abundance at the phylum level and enriching *Bacillus* while reducing *Clostridium sensu stricto 1* at the genus level. Functional prediction based on PICRUSt2 indicated an enhanced metabolic potential of the gut microbiota, with inferred upregulation of pathways related to carbohydrate transport and metabolism (e.g., ABC transporters) and intestinal enzymatic activities. Collectively, BS-FZU103 is associated with metabolic modulation, promoting *M. salmoides* growth through gut microbiota remodeling, hepatic antioxidant fortification, and targeted augmentation of carbohydrate utilization efficiency.

## 1. Introduction

Sustainable aquaculture is fundamental to the FAO’s Blue Transformation strategy and plays an indispensable role in ensuring global food security [1]. However, aquaculture intensification and concomitant high-density practices, despite boosting productivity, elevate risks of disease outbreaks and environmental pollution, thereby challenging sustainability [2,3,4]. This challenge is particularly acute in the farming of largemouth bass (*Micropterus salmoides*), where high-density rearing has historically fostered an excessive dependence on antibiotics for disease control [5,6,7,8]. The long-term use of antibiotics in intensive aquaculture has accelerated the development of antimicrobial resistance (AMR), which has now become a major global public health threat [9,10,11]. Given the limitations on antibiotic use, there is an urgent industry need to find safe and effective alternatives for species such as *M. salmoides*, which rely on high-density farming. In this context, probiotics have emerged as a safe alternative to antibiotics, presenting a key breakthrough for the green aquaculture of *M. salmoides.*

Probiotics are defined as “live microorganisms that confer a health benefit on the host” [12]. They primarily function in the gut to improve host health [13]. In aquaculture, the role of probiotics is not limited to the animal’s intestinal tract; they can also indirectly promote health by improving water quality, making them a potential antibiotic-alternative strategy [14]. Currently, probiotics are widely used in aquaculture [14]. Extensive research has demonstrated that *Bacillus* and *Lactobacillus* spp., among the most prevalent probiotics used in aquaculture, enhance growth performance, digestive function, and immunity in diverse aquatic species, including fish, shrimp, and crabs [15,16,17]. For instance, *Lactobacillus rhamnosus* GG (LGG) demonstrates multifaceted probiotic effects. Sewaka et al. [18] investigated the effects of LGG supplementation in the diet of red tilapia (*Oreochromis* spp.). The study found that LGG significantly improved the specific growth rate (SGR), and average daily gain (ADG), effectively promoting the growth of *Oreochromis* spp. Similarly, Noshair et al. [19] demonstrated that LGG supplementation enhances growth, boosts immunity, and improves disease resistance in *Nile tilapia* (*Oreochromis niloticus*). Despite this evidence, current aquaculture probiotic research remains predominantly focused on single-strain evaluations. A significant gap exists in comparative studies that systematically assess the relative efficacy of different probiotic strains within the same host species. This limitation impedes the identification of optimal probiotics for specific aquaculture applications [20,21]. In addition, how these probiotic-driven changes in the intestinal microbiota enhance the antioxidant capacity of fish, and how they promote the activity of particular digestive enzymes (e.g., amylase, protease, lipase) to improve nutrient digestion and absorption, still require further investigation [14].

This study was designed to comparatively evaluate the efficacy of three probiotic candidates (LGG, *Lactobacillus plantarum* FZU310 (LP-FZU310), and *Bacillus subtilis* FZU103 (BS-FZU103)) in *M. salmoides*, in order to test the hypothesis that BS-FZU103 would outperform *Lactobacillus* strains. Our objectives were to assess their effects on growth performance, key health indicators, and gut microbiota, and to elucidate the underlying growth-promotion mechanisms by deciphering gut microbiota-host physiological interplay. To mechanistically achieve these objectives, we employed an integrated approach utilizing high-throughput 16S rRNA gene sequencing and PICRUSt2-based analysis to comprehensively profile gut microbial shifts and their predicted functional potential, thereby enabling the exploration of correlations between specific microbiota alterations and host physiological responses.

## 2. Materials and Methods

### 2.1. Experimental Materials

Healthy juvenile largemouth bass (*M. salmoides*; initial weight 35 ± 1 g) were obtained from the Marine Center of Fuzhou University (Fuzhou, Fujian, China) and acclimatized to laboratory conditions for two weeks prior to the trial. This study strictly adhered to the international guidelines and principles of animal experimentation to ensure that the suffering of experimental animals was minimized. Before the experiment began, we obtained the approval of the Animal Ethics Committee of Fuzhou University (approval number: FZU-IFST-202226). Initial mean body weight of each group: 35.14 ± 0.61 g (Control group), 35.16 ± 0.68 g (LGG group), 35.11 ± 0.65 g (LP group), 35.26 ± 0.56 g (BS group). Initial mean length of each group: 13.2 ± 0.52 cm (Control group), 13.05 ± 0.42 cm (LGG group), 13.26 ± 0.29 cm (LP group), 13.17 ± 0.38 cm (BS group). Fish were fed a commercially formulated basal diet (Fujian Tianma Science and Technology Group Co., Ltd., Fuqing, China) with a composition of 50% crude protein and 6% crude lipid (dry weight basis). Three probiotic strains were utilized: LGG, LP-FZU310, and BS-FZU103. All strains were sourced from the Institute of Food Science and Technology, College of Biological Science and Technology, Fuzhou University (Fuzhou, China). Strain viability was confirmed via standard plate counting on appropriate media (de Man, Rogosa and Sharpe (MRS) agar medium for LGG and LP-FZU310, Lysogeny Broth (LB) agar medium for BS-FZU103) prior to feed preparation. All chemicals and kits for biochemical analyses (digestive enzymes, antioxidant enzymes, liver function markers, malondialdehyde (MDA)) were obtained from Nanjing Jiancheng Bioengineering Institute (Nanjing, Jiangsu, China) and were of analytical grade.

### 2.2. Probiotic Preparation and Feed Formulation

BS-FZU103 was cultured aerobically in sterile LB medium. An inoculum (1% *v*/*v*; initial OD_600_ = 0.5) was incubated at 37 °C with shaking (180 rpm) for 12 h to reach late-logarithmic phase [22]. LGG and LP-FZU310 were cultured anaerobically in sterile MRS medium at 37 °C under static conditions [23]. Bacterial cells from each culture were harvested by centrifugation (5000× *g* rmp, 10 min, 4 °C). The pellets were washed twice with sterile phosphate-buffered saline (PBS; pH 7.4) to remove residual culture medium components. The final cell pellets were resuspended in sterile distilled water. The optical density (OD_600_) of the suspensions was measured and adjusted to achieve the target concentration for feed supplementation. The probiotic suspensions were evenly sprayed onto the surface of the basal diet pellets using an atomizer, ensuring uniform distribution. The coated feeds were then air-dried at room temperature (25 ± 2 °C) under sterile laminar flow for approximately 4–6 h. Viable probiotic count in the coated diet was quantified immediately after preparation using the plate count method on LB agar (BS-FZU103) or MRS agar (LGG, LP-FZU310). The final concentration for all probiotic-supplemented diets was confirmed to be 1 × 10^8^ CFU/g feed. Diets were prepared weekly and stored at 4 °C in sealed sterile containers to maintain probiotic viability until use. Control diet viability was confirmed as negligible.

### 2.3. Experimental Design and Feeding Management

Fish (*n* = 40) were randomly distributed into four groups (*n* = 10 per group):

Control: Fed basal diet coated with sterile saline.

LGG Group: Fed basal diet coated with LGG (1 × 10^8^ CFU/g feed).

LP Group: Fed basal diet coated with LP-FZU310 (1 × 10^8^ CFU/g feed).

BS Group: Fed basal diet coated with BS-FZU103 (1 × 10^8^ CFU/g feed).

The concentration of probiotics in the feed is referenced from Ruiz et al. [24]. Fish were hand-fed to apparent satiation twice daily (at 09:00 and 18:00) for 30 consecutive days. To ensure consistency and reduce bias, the feeding procedure was standardized: all fish were fed by the same person following a fixed protocol. The feeder observed fish feeding behavior closely and stopped offering feed when the majority of fish lost interest and moved away from the feeding area. The total amount of feed consumed per group per day was recorded to verify that feeding levels were comparable. *M. salmoides* were divided into four groups, each with three independent tanks equipped with separate aeration devices, inlets, and outlets; the 12 tanks shared one recirculating aquaculture system (RAS), and water was evenly distributed after passing through a biofilter and a sedimentation tank. Tanks were filled with municipal tap water dechlorinated by vigorous aeration for ≥48 h prior to use. Water quality parameters were monitored daily: temperature was maintained at 24.0 ± 0.5 °C using heaters/chillers, dissolved oxygen (DO) > 5.0 mg/L via continuous aeration, pH 7.2–7.5, total ammonia nitrogen (TAN) < 0.05 mg/L, and nitrite (NO_2_^−^) < 0.02 mg/L. Partial water exchanges (20–30% volume) were performed every third day to maintain water quality. A 12 h:12 h light/dark photoperiod was maintained.

### 2.4. Sample Collection and Processing

After the 30-day feeding period, fish were fasted for 24 h. All fish per replicate were individually weighed to determine final body weight (FBW). Key growth performance indices were calculated as follows [25]:

Weight gain rate (WGR, %) = [(FBW − Initial weight)/Initial weight] × 100

Specific growth rate (SGR, %) = [(Ln FBW − Ln Initial weight)/Feeding days] × 100

Feed conversion ratio (FCR) = Total dry feed fed (g)/Total weight gain (g)

Condition factor (CF, g/cm^3^) = [Body weight (g)/(Total length (cm))^3^] × 100

Fish were fasted for 24 h to eliminate the postprandial effects on blood parameters. Subsequently, fish were anesthetized using a buffered solution of eugenol (50 mg/L). Blood was collected from the caudal vasculature using heparinized syringes. Blood was allowed to clot at 4 °C for 2–4 h, then centrifuged (3000× *g* rmp, 10 min, 4 °C). Serum aliquots were stored at −80 °C. Liver and the entire intestine were aseptically dissected immediately after euthanasia. Tissues were rinsed in ice-cold saline (0.86%), blotted dry, snap-frozen in liquid nitrogen, and stored at −80 °C until analysis. Frozen tissues were weighed and homogenized (1:9 *w*/*v*, or as per specific assay kit instructions) in ice-cold physiological saline (0.86%) or appropriate homogenization buffer (e.g., 0.1 M phosphate buffer, pH 7.4) using a pre-chilled homogenizer. Homogenate was centrifuged (2500× *g* rmp, 10 min, 4 °C). The resulting supernatant was collected, aliquoted, and stored at −80 °C for subsequent biochemical assays. Protein concentration in the supernatant was determined using the Bradford or BCA method for normalization where required.

### 2.5. Biochemical Assays

Serum alanine aminotransferase (ALT), aspartate aminotransferase (AST) and alkaline phosphatase (ALP) were determined spectrophotometrically using commercial assay kits strictly following the manufacturer’s protocols (Nanjing Jiancheng Bioengineering Institute, Nanjing, China) [26,27,28]. Liver homogenate supernatants were assayed for superoxide dismutase (SOD), glutathione peroxidase (GPx) activities and MDA concentration. Intestinal homogenate supernatants were assayed for amylase, protease, lipase activities. All enzyme activities and MDA concentrations were normalized to tissue protein content determined via the Bradford assay and expressed accordingly (e.g., U/mg protein and nmol/mg protein).

### 2.6. Gut Microbiota Analysis

Microbial genomic DNA was extracted from intestinal contents (*n* = 8 per group) using the QIAamp PowerFecal Pro DNA Kit (Hilden, Germany) according to the manufacturer’s protocol. DNA concentration and purity were assessed using spectrophotometry (NanoDrop; Thermo Fisher Scientific, Wilmington, DE, USA; A260/A280 > 1.8) and agarose gel electrophoresis. The hypervariable V3-V4 region of the bacterial 16S rRNA gene was amplified using universal primers 338F (5′-ACTCCTACGGGAGGCAGCAG-3′) and 806R (5′-GGACTACHVGGGTWTCTAAT-3′). PCR products were purified, quantified, and pooled in equimolar ratios. Paired-end sequencing was performed on an Illumina Nextseq2000 platform (Illumina, San Diego, CA, USA) according to the standard protocols by Majorbio Bio-Pharm Technology Co., Ltd. (Shanghai, China). Raw sequence data were processed using QIIME2 (2022.2) or Mothur (v1.30.1). Steps included demultiplexing, quality filtering (based on Q-scores), denoising (DADA2 or UNOISE3) to infer exact amplicon sequence variants (ASVs), chimera removal, and merging of paired-end reads. Taxonomic assignment was performed against a reference database (SILVA, Greengenes). Alpha diversity and beta diversity analyses were conducted. Functional potential was predicted using PICRUSt2 (v2.2.0) or Tax4Fun2 (R-3.3.1) based on the KEGG database.

### 2.7. Statistical Method

All data are presented as mean ± standard deviation (SD). Statistical analyses were performed using SPSS software (version 26.0, IBM Corp., Armonk, NY, USA). Normality (Shapiro–Wilk test) and homogeneity of variance (Levene’s test) were confirmed. One-way analysis of variance (ANOVA) followed by Tukey’s honestly significant difference post hoc test was used to determine significant differences (*p* < 0.05) among treatment groups for growth, biochemical, and enzyme data. Beta diversity differences were assessed using permutational multivariate analysis of variance based on Bray–Curtis distances. Spearman’s rank correlation analysis was performed to identify significant correlations between dominant microbial genera and host physiological parameters. Differences at *p* < 0.05 were considered statistically significant.

## 3. Results

### 3.1. Effects of LGG, LP-FZU310 and BS-FZU103 on the Growth of M. salmoides

**Figure 1:**
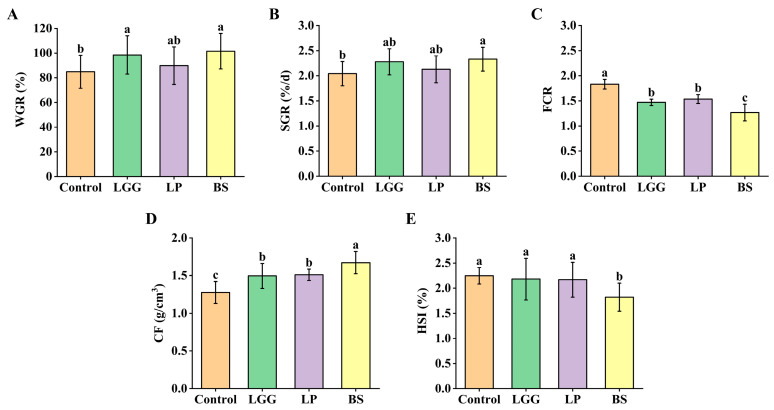
Effects of LGG, LP-FZU310 and BS-FZU103 on the growth performance of *M. salmoides*. (**A**) WGR. (**B**) SGR. (**C**) FCR. (**D**) CF. (**E**) HSI. Values are presented as mean ± SD. Different lowercase letters indicate significant differences between groups (*p* < 0.05), while the same lowercase letters indicate no significant differences between groups (*p* > 0.05).

The effects of dietary supplementation with LGG, LP-FZU310 and BS-FZU103 on the growth parameters of *M. salmoides* are summarized in Figure 1. BS-FZU103 demonstrated the most pronounced growth-promoting effects. Compared to the control group, the BS group exhibited significant differences in both WGR and SGR (*p* < 0.05 for each). The LGG group showed a significant improvement in WGR (*p* < 0.05), although the increase in SGR did not reach statistical significance. In contrast, the LP-FZU310 group did not significantly alter either WGR or SGR compared to controls.

Regarding feed utilization efficiency, all probiotic-supplemented groups (LGG, LP-FZU310, BS-FZU103) displayed a significant reduction in FCR (*p* < 0.05). The most substantial reduction was observed in the BS-FZU103 group, indicating superior feed efficiency for this strain. Furthermore, all probiotic groups showed a significant difference in CF compared with the control group (*p* < 0.05), reflecting improved body conformation and nutritional status. Notably, only the BS-FZU103 group showed a significant difference in the hepatosomatic index (HSI) compared with the control group (*p* < 0.05).

### 3.2. Effects of LGG, LP-FZU310 and BS-FZU103 on Hepatic Antioxidant Status

**Figure 2:**
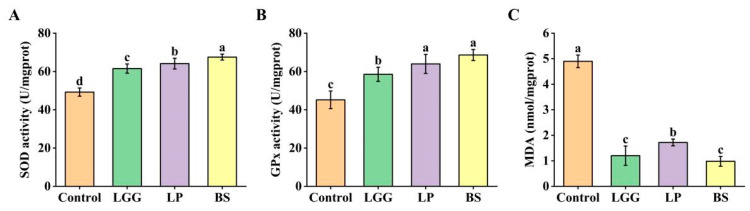
Effects of LGG, LP-FZU310 and BS-FZU103 on liver enzyme in *M. salmoides*. (**A**) Effect of probiotics on SOD. (**B**) Influence of probiotics on GPx. (**C**) Effect of probiotics on MDA. Values are presented as mean ± SD. Different lowercase letters indicate significant differences between groups (*p* < 0.05), while the same lowercase letters indicate no significant differences between groups (*p* > 0.05).

Probiotic supplementation significantly modulated the hepatic antioxidant defense system, with BS-FZU103 inducing the most robust enhancement (Figure 2). SOD activity increased significantly from 49.24 U/mg prot in the control group to 61.52 U/mg prot (LGG; +24.94%), 64.12 U/mg prot (LP-FZU310; +30.22%), and 67.51 U/mg prot (BS-FZU103; +37.1%) (Figure 2A). Similarly, GPx activity rose significantly from 45.18 U/mg prot (Control) to 58.51 U/mg prot (LGG; +29.5%), 63.96 U/mg prot (LP-FZU310; +41.57%), and 68.62 U/mg prot (BS-FZU103; +51.88%) (Figure 2B). Concurrently, the level of MDA, a key indicator of lipid peroxidation and oxidative damage, was markedly reduced in all probiotic groups compared to the control (4.9 nmol/mg prot) (Figure 2C). Reductions in MDA were observed to 1.2 nmol/mg prot (LGG; −75.48%), 1.72 nmol/mg prot (LP-FZU310; −64.9%), and 0.98 nmol/mg prot (BS-FZU103; −80.00%). Collectively, these results demonstrate that BS-FZU103 supplementation most effectively strengthened the hepatic antioxidant defense system, evidenced by the highest elevations in SOD (U/mg prot) and GPx (U/mg prot) activities and the most substantial reduction in MDA (nmol/mg prot) levels, with all values normalized to protein content.

### 3.3. Effects of LGG, LP-FZU310 and BS-FZU103 on Intestinal Digestive Enzyme Activities

**Figure 3:**
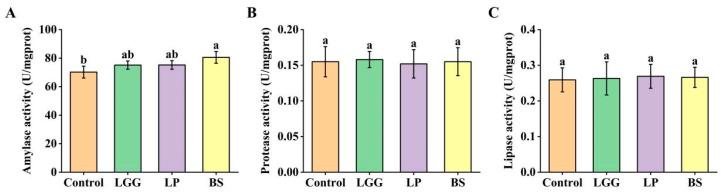
Effects of LGG, LP and BS on digestive enzymes in *M. salmoides*. (**A**) Amylase activity. (**B**) Protease activity. (**C**) Lipase activity. Values are presented as mean ± SD. Different lowercase letters indicate significant differences between groups (*p* < 0.05), while the same lowercase letters indicate no significant differences between groups (*p* > 0.05).

The effects of probiotic supplementation on key intestinal digestive enzyme activities differed among the strains tested (Figure 3). BS-FZU103 improved carbohydrate digestion, as reflected by a significant increase (*p* < 0.05) in intestinal amylase activity. (+14.7% vs. control). In contrast, no significant alterations in amylase activity were observed in the LGG or LP groups compared to the control (*p* > 0.05). Furthermore, the activities of protease and lipase remained largely unaffected across all probiotic-supplemented groups relative to the control group, indicating no broad-spectrum enhancement of protein or lipid digestion by these probiotics under the experimental conditions. The specific increase in amylase activity by BS-FZU103 highlights its potential role in augmenting carbohydrate utilization pathways in *M. salmoides*.

### 3.4. Effects of LGG, LP-FZU310 and BS-FZU103 on Serum Biomarkers

**Figure 4:**
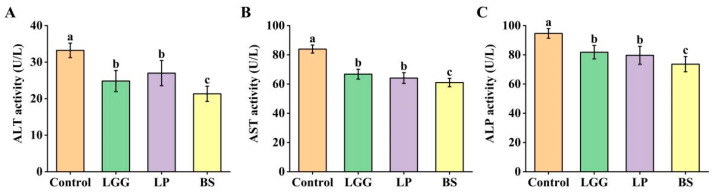
Effects of probiotics on serum enzymes in *M. salmoides*. (**A**) ALT activity. (**B**) AST activity. (**C**) ALP activity. Values are presented as mean ± SD. Different lowercase letters indicate significant differences between groups (*p* < 0.05), while the same lowercase letters indicate no significant differences between groups (*p* > 0.05).

Serum biomarker analysis revealed significant improvements in indicators of liver health following probiotic supplementation (Figure 4). Compared to the control group, all probiotic groups (LGG, LP-FZU310, BS-FZU103) exhibited significant reductions (*p* < 0.05) in the activities of ALT (reduced by 35.8% in BS group), AST (reduced by 27.27% in BS group), and ALP (reduced by 22.21% in BS group). These reductions collectively suggest enhanced hepatocellular integrity and reduced liver stress or damage. Notably, the BS group consistently demonstrated the most pronounced ameliorative effects on these serum biomarkers compared to both the control and the other probiotic groups.

### 3.5. BS-FZU103 Administration Induces Distinct Gut Microbiota Remodeling

**Figure 5:**
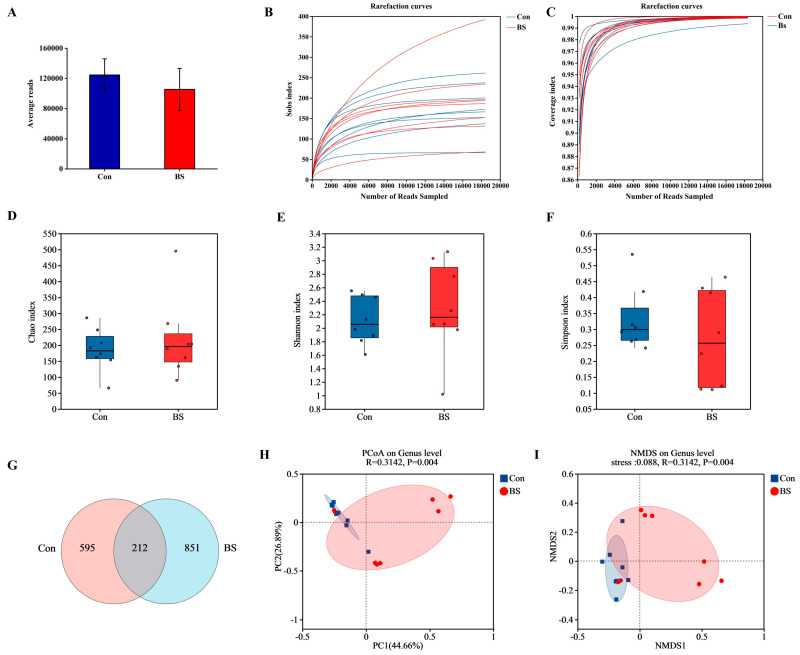
Differences in microbiota among different groups. (**A**) Average reads. (**B**) Rarefaction curves. (**C**) Sequencing depth index. (**D**–**F**) The impact of BS-FZU103 on α-diversity index; (**D**) Chao index. (**E**) Shannon index. (**F**) Simpson index. (**G**) Venn Diagram. (**E**,**F**) The impact of BS on β-diversity; (**H**) PCoA. (**I**) NMDS analysis. Values are presented as mean ± SD, *n* = 8.

**Figure 6:**
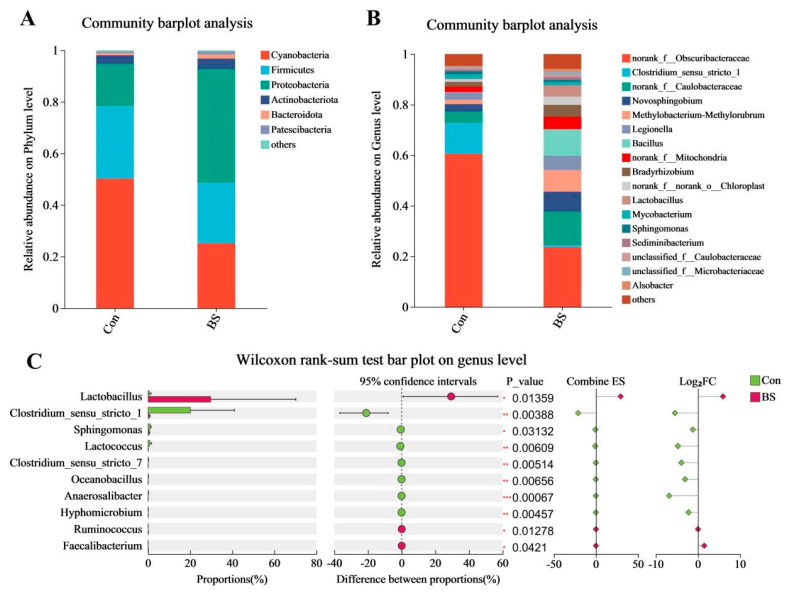
Effects of BS-FZU103 at the phylum and genus levels. (**A**) Impact of BS-FZU103 at the phylum level. (**B**) Impact of BS at the genus level. (**C**) Wilcoxon rank sum analysis at the genus level. * *p* < 0.05, ** *p* < 0.01, *** *p* < 0.001.

The impact of BS-FZU103 supplementation on the gut microbiota composition of *M. salmoides* was profound (Figure 5 and Figure 6). Figure 5A shows the average reads of each group (control: 124,675 ± 21,365, BS: 105,511 ± 27,817), ensuring the reliability and accuracy of the analysis results. The rarefaction coverage eventually plateaus, indicating that the sequencing depth is sufficient and the sample data have been standardized (Figure 5B,C). Prior to analysis, sequence data were rarefied to an equal depth to ensure comparability of alpha diversity. Alpha-diversity analysis (Chao, Shannon, and Simpson indices; Figure 5D–F) indicated no significant alteration in species richness or evenness between the BS group (BS) and the control group (Con). Venn analysis of Amplicon Sequence Variants (ASVs) highlighted distinct community structures, identifying 595 ASVs unique to the control, 851 ASVs exclusive to the BS group, and only 212 ASVs shared between the two groups (Figure 5G). Beta-diversity analysis, visualized via Principal Coordinates Analysis (PCoA; Figure 5H) and Non-Metric Multidimensional Scaling (NMDS; Figure 5I), confirmed a significant and clear separation (*p* < 0.05, PERMANOVA) between the gut microbial communities of the control and BS groups.

Taxonomic profiling revealed substantial compositional shifts. At the phylum level (Figure 6A), *Cyanobacteria* dominated the control microbiota. Remarkably, BS-FZU103 supplementation profoundly altered this structure, leading to *Proteobacteria* becoming the dominant phylum. At the genus level (Figure 6B), a dramatic restructuring was observed. *Clostridium sensu stricto 1* overwhelmingly prevailed in the control group but experienced a sharp decline in relative abundance within the BS group. Conversely, *Bacillus* emerged as the top-ranking genus in the BS-FZU103 supplemented fish. Further analysis (Figure 6C) confirmed that BS-FZU103 supplementation significantly enriched the relative abundance of *Lactobacillus* while simultaneously suppressing *Clostridium sensu stricto 1*.

### 3.6. Correlation Analysis of Between Gut Microbiota and Host Physiological Parameters

**Figure 7:**
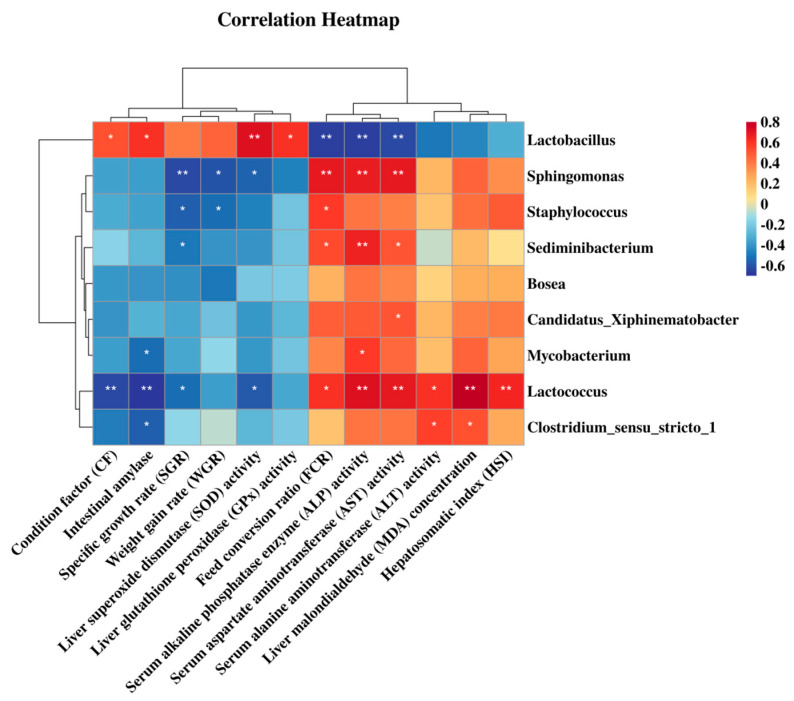
Spearman analysis of gut microbiota and host physiological parameters. * *p* < 0.05, ** *p* < 0.01.

Spearman rank correlation analysis, visualized as a heatmap (Figure 7), revealed significant associations between key gut microbial genera and host physiological parameters. The relative abundance of *Lactobacillus* exhibited strong positive correlations (*p* < 0.05) with CF, intestinal amylase activity, hepatic SOD and GPx activities. Conversely, *Lactobacillus* abundance correlated negatively (*p* < 0.01) with FCR and the serum biomarkers ALT and AST. In contrast, FCR, ALP, and AST levels showed positive correlations (*p* < 0.05) with genera such as *Sphingomonas*, *Sediminibacterium*, and *Lactococcus*. *Lactococcus* abundance also correlated positively (*p* < 0.05) with serum ALT, hepatic MDA levels, and HSI. These correlations suggest potential functional links between specific microbial taxa and host growth performance, metabolic efficiency, digestive function, antioxidant status, and liver health.

### 3.7. BS-FZU103 Administration Alters the Metabolic Potential of the Intestinal Microbiota

**Figure 8:**
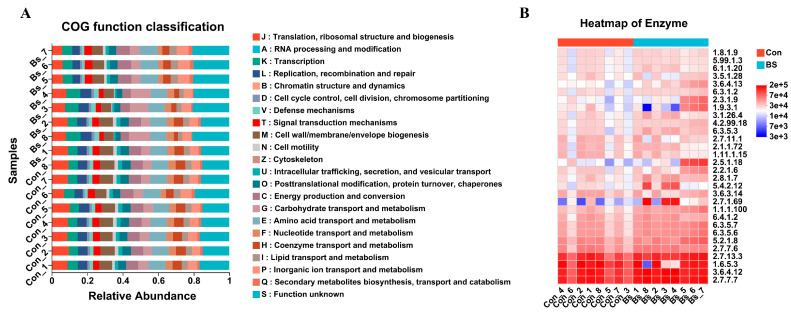
The effect of BS on metabolic pathways and intestinal enzymes. (**A**) Effects of BS on the metabolic pathways of *M. salmoides*. (**B**) Effects of BS on the intestinal enzymes of *M. salmoides*.

The metabolic potential of the gut microbiota was inferred using PICRUSt2. This predictive analysis suggested that BS-FZU103 supplementation altered the putative functional landscape (Figure 8A). Specifically, the BS group displayed a decrease in predicted genes for “Translation, ribosomal structure and biogenesis” and an increase for “Carbohydrate transport and metabolism” compared to the control. Similarly, predicted enzyme abundances (Figure 8B) were suggestive of elevated activity in pathways for carbohydrate metabolism, amino acid/peptide processing, and redox balance in the BS group. It should be noted that these are computational predictions.

**Figure 9:**
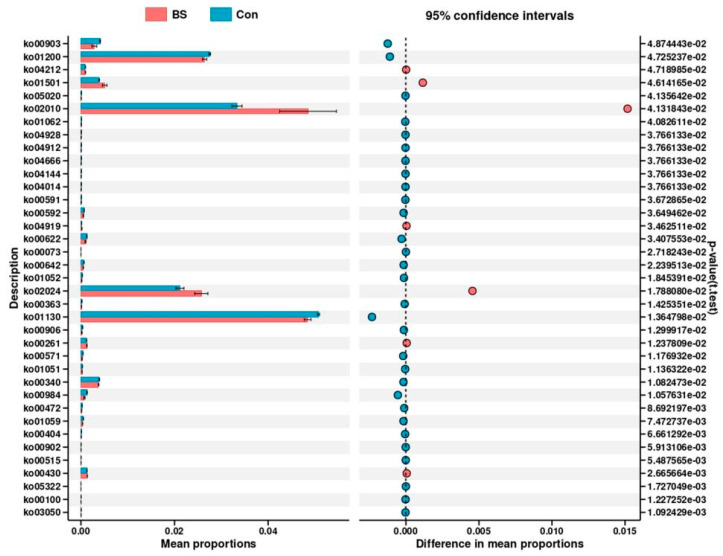
Differential analysis of metabolic pathways in *M. salmoides*.

**Figure 10:**
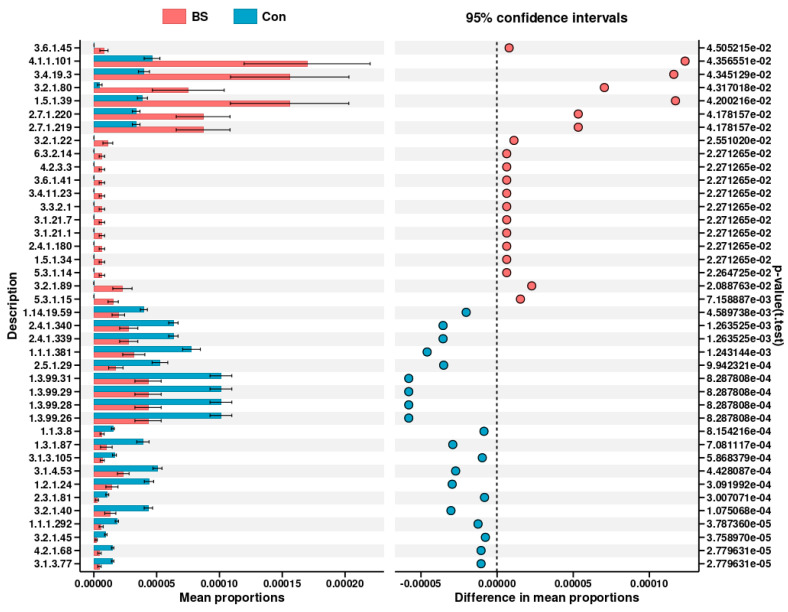
Differential analysis of gut enzyme levels in *M. salmoides*.

Direct assessment of differentially abundant metabolic pathways (Figure 9) further highlighted specific functional changes. The BS-FZU103 group showed significant enrichment in pathways including the ATP-binding cassette (ABC) transporters (ko02010), Quorum sensing (ko02024), β-Lactam resistance (ko01501), Limonene degradation (ko00903), and Carbon metabolism (ko01200). Analysis of differential enzyme abundances predicted from the metagenome (Figure 10) revealed that BS-FZU103 supplementation significantly increased the abundance of enzymes involved in key metabolic processes compared to the control. Notably enhanced enzymes included: Malolactic enzyme (EC 4.1.1.101), Pyroglutamyl-peptidase I (EC 3.4.19.3), Xylanase (EC 3.2.1.80 and EC 3.2.1.89), Formate dehydrogenase (EC 1.5.1.39), Phosphofructokinase 2 (EC 2.7.1.220), Phosphofructokinase 1 (EC 2.7.1.219), and Fructan β-fructosidase (EC 3.2.1.22). This pattern suggests enhanced capabilities in carbohydrate metabolism (xylanase, fructosidase, phosphofructokinases), amino acid/peptide processing (malolactic enzyme, pyroglutamyl-peptidase), and detoxification/redox balance (formate dehydrogenase).

## 4. Discussion

The aquaculture industry increasingly recognizes probiotics as viable alternatives to antibiotics, driven by concerns over antibiotic resistance and environmental contamination [29,30,31]. Probiotics offer sustainable strategies to enhance fish health and growth performance [32,33,34]. Their beneficial effects stem from intestinal colonization, fostering host-microbe symbiosis, and the production of bioactive compounds like short-chain fatty acids, enzymes, and exopolysaccharides, which bolster host immunity and reduce disease incidence [35,36,37,38]. In the present study, dietary supplementation with LGG, LP-FZU310 and BS-FZU103 differentially improved growth parameters in *M. salmoides*. Notably, BS-FZU103 demonstrated superior efficacy, significantly enhancing SGR and CF compared to controls. Xue et al. [39] fed *Pelteobagrus fulvidraco* with 10^6^ cfu/g of *Bacillus amyloliquefaciens* for four weeks, which significantly enhanced the SGR, similar to our research findings. This indicates that BS-FZU103 facilitates more efficient conversion of dietary nutrients into energy for growth and development in *M. salmoides*. The enhanced performance might be tentatively ascribed to the potential inherent robustness of BS, including its putative stress resistance, metabolic versatility, and environmental compatibility, which could theoretically facilitate its adaptation to the intestinal milieu [40]. Notably, the decrease in HSI suggests a potential improvement in liver health, as a lower HSI often indicates reduced hepatic lipid accumulation or lower metabolic burden on the liver in fish, which may correspond to enhanced liver function and overall metabolic efficiency in *M. salmoides* [41]. These findings underscore BS-FZU103 as the most effective probiotic among those tested for promoting *M. salmoides* growth. This aligns with studies reporting *Bacillus* spp. improving growth in other fish species like Pagrus major [42], confirming the genus’s potent growth-promoting capabilities, with BS-FZU103 exhibiting particular effectiveness in *M. salmoides*.

The liver serves as a critical hub for detoxification and metabolic regulation, where oxidative stress can significantly impair function [43]. BS-FZU103 induced the most potent upregulation of key hepatic antioxidant enzymes, SOD (+37.1%) and GPx (+51.88%), coupled with the most dramatic reduction in the lipid peroxidation marker MDA (−80.0%). This triad of effects signifies a profound strengthening of the cellular antioxidant defense network. SOD catalyzes the dismutation of superoxide radicals to hydrogen peroxide [44], while GPx detoxifies hydrogen peroxide and lipid hydroperoxides using glutathione [45], acting synergistically to mitigate oxidative damage. The dramatic decline in MDA levels confirms reduced oxidative insult to hepatic membranes. Crucially, the significantly improved serum biomarkers (ALT −35.8%, AST −27.27%, ALP −22.21%) in the BS group provide compelling evidence of enhanced hepatocellular integrity and reduced liver stress or damage, linking the antioxidant boost directly to improved organ health status. This superior hepatic antioxidant enhancement by BS-FZU103 establishes a state of enhanced host-microbe mutualism critical for overall health and resilience.

Intestinal digestive enzyme activity is a key indicator of gut health and nutrient utilization potential [46]. Probiotics can enhance this process by modulating host enzyme activity or through microbial metabolite production [47,48]. Unlike some probiotics reported to induce broad-spectrum increases in multiple digestive enzymes [49,50,51], BS-FZU103 elicited a specific and significant enhancement of amylase activity (+14.7%). This targeted modulation suggests an optimized strategy for *M. salmoides*. Given the carnivorous nature of *M. salmoides*, the specific enhancement of carbohydrate digestion suggests BS-FZU103 may improve the utilization of dietary carbohydrates or plant-derived ingredients in formulated feeds, potentially contributing to the observed superior feed efficiency (reduced FCR).

Serum biomarkers such as ALT, AST, and ALP reflect liver integrity and function. Probiotic modulation of these enzymes is well-documented [52,53,54]. Consistent with this literature, dietary LGG, LP-FZU310, and BS-FZU103 significantly reduced serum levels of AST (27.27%), ALT (35.8%), and ALP (22.21%) in *M. salmoides*. These reductions indicate improved liver health and reduced hepatocellular damage. BS-FZU103 again demonstrated the strongest regulatory effect on these serum biomarkers compared to the other probiotics.

The gut microbiota plays a fundamental role in nutrient metabolism, barrier function, and host immunity [55,56]. Probiotics can restructure microbial communities and restore well-being [57]. BS-FZU103 administration induced a significant structural reorganization of the *M. salmoides* gut microbiota, as unequivocally demonstrated by clear separation in beta-diversity analyses (PCoA, NMDS; *p* < 0.05), despite unaltered alpha diversity. This reorganization manifested as a dramatic shift from *Cyanobacteria* dominance (control) to *Proteobacteria* dominance (BS group) at the phylum level. While *Proteobacteria* expansion is often viewed as a potential indicator of dysbiosis [58], the observed increase coincided with a clear alleviation of oxidative stress (lower MDA). This implies that the *Proteobacteria* bloom in the BS-FZU103 group may reflect harmless or even beneficial genera whose metabolic output contributed to the improved redox status, rather than an overt pathogenic signature. At the genus level, BS-FZU103 treatment led to a significant shift, with the predominance of *Clostridium sensu stricto 1* in the control group being substantially decreased. This change was accompanied by a notable enrichment of the *Bacillus* genus, to which the supplemented BS-FZU103 strain belongs. The altered microbial landscape, marked by an increase in *Bacillus* and a reduction in *Clostridium*, may facilitate the competitive exclusion of potential pathogens, potentially through mechanisms such as niche competition and antimicrobial production [59]. The concurrent significant enrichment of beneficial *Lactobacillus* further contributes to a healthier microbial community structure [60]. It has been reported that *Lactobacillus*, as a well-recognized probiotic, can produce other organic acids such as acetic acid and propionic acid [61]. These short-chain fatty acids (SCFAs) help regulate the gut microbiota and play an important role in health [62]. Crucially, Spearman correlation analysis revealed significant associations between these microbial shifts and host physiology. The positive correlation of *Lastobacillus* abundance with CF, amylase activity, SOD, and GPx, and its negative correlation with FCR, ALT, and AST, collectively support a functional link between BS-FZU103-induced microbiota remodeling and the concurrent improvements in growth, digestive efficiency, antioxidant status, and liver health. While these robust associations do not establish causation, they provide a strong rationale for future studies to investigate the mechanistic role of specific microbial taxa, such as *Lactobacillus*, in mediating the benefits of BS-FZU103.

Numerous studies have shown that probiotics can promote the digestion and absorption of nutrients by modulating metabolic functions [63]. Falcinelli et al. [64] added LGG to the diet and investigated its effects on the metabolism of zebrafish, finding that the transcription of genes related to cholesterol and triglyceride (TAG) metabolism was reduced. Zhang et al. [65] explored the effects of *Lactobacillus* casei YYL3 (Lc) on the metabolism of Channel Catfish and found that Lc upregulated lipid metabolism, metabolism of other amino acids, metabolism of terpenoids and polyketides, biodegradation and metabolism of xenobiotics, and nucleotide metabolism. Beyond taxonomic changes, BS-FZU103 significantly altered the predicted functional potential of the gut microbiota. Analyses indicated an upregulation of pathways central to nutrient acquisition and metabolism, particularly “Carbohydrate transport and metabolism” and the ATP-binding cassette (ABC) transporter system (ko02010). ABC transporters are vital for importing nutrients and exporting toxins, suggesting enhanced substrate uptake and detoxification capabilities. Furthermore, BS-FZU103 supplementation significantly increased the predicted abundance of key enzymes involved in carbohydrate metabolism (Xylanase [EC 3.2.1.80/89], Fructan β-fructosidase [EC 3.2.1.22], Phosphofructokinases [EC 2.7.1.219/220]), amino acid/peptide processing (Malolactic enzyme [EC 4.1.1.101], Pyroglutamyl-peptidase I [EC 3.4.19.3]), and redox balance (Formate dehydrogenase [EC 1.5.1.39]). These functional enhancements align perfectly with the observed increase in intestinal amylase activity and the specific improvement in carbohydrate utilization, and likely contribute to the overall metabolic efficiency and growth promotion. The enrichment of pathways like Quorum sensing (ko02024), β-Lactam resistance (ko01501), and Limonene degradation (ko00903) also suggests potential modulation of microbial communication, stress responses, and xenobiotic metabolism. It is important to note that these predictions are based on PICRUSt2 analysis. This method, while powerful, does not provide direct measurements of these pathways. Therefore, future studies with direct functional assays would be necessary to validate these predictions. In addition, the detection of β-lactam resistance-related genes is a common observation in gut microbiome studies and may reflect a general stress response or intrinsic resistance of the commensal microbiota, rather than conferring a direct phenotypic resistance threat. Nonetheless, the ecological implications of such enrichment warrant consideration in the context of probiotic applications.

## 5. Conclusions

In this study, we demonstrate that *B. subtilis* FZU103 is a highly effective probiotic for enhancing largemouth bass (*M. salmoides*) growth and health by orchestrating gut microbiota remodeling, hepatic antioxidant fortification, and targeted digestive modulation. Specifically, BS-FZU103 significantly elevated SGR and CF, outperforming other probiotics. BS-FZU103 was associated with shifts in the gut microbial structure, increasing *Proteobacteria* and *Bacillus* while suppressing *Clostridium sensu stricto 1*. Predictive functional analysis (PICRUSt2) linked these taxonomic shifts to an enhancement in carbohydrate metabolism-related pathways, such as ABC transporters and xylanase activity. Antioxidant levels significantly increased (SOD and GPx), effectively reducing the oxidative damage marker MDA, while also improving liver function. Digestively, BS-FZU103 selectively increased amylase activity in *M. salmoides*, indicating enhanced carbohydrate utilization. However, it is important to consider that the effects of BS-FZU103 on amylase activity might be influenced by dietary composition and culture conditions. Further studies are needed to investigate how variations in diet, such as the proportion of carbohydrates, proteins, and lipids, as well as culture parameters like temperature, dissolved oxygen, and stocking density, could modulate the response of *M. salmoides* to BS-FZU103 supplementation. Collectively, BS-FZU103 acts as a multifaceted metabolic modulator, driving growth through microbiota-host crosstalk, systemic antioxidant enhancement, and nutrient efficiency optimization. These findings position BS-FZU103 as a promising candidate for sustainable *M. salmoides* aquaculture. Future research should focus on validating the efficacy and optimal application strategies of BS-FZU103 in large-scale aquaculture settings, and further elucidate the precise molecular crosstalk between this probiotic, the remodeled gut microbiome, and host metabolic pathways governing growth and health.

## Data Availability

The original contributions presented in this study are included in the article. Further inquiries can be directed to the corresponding authors.
